# Effects and Clinical Significance of GII.4 Sydney Norovirus, United States, 2012–2013 

**DOI:** 10.3201/eid1908.130458

**Published:** 2013-08

**Authors:** Eyal Leshem, Mary Wikswo, Leslie Barclay, Eric Brandt, William Storm, Ellen Salehi, Traci DeSalvo, Tim Davis, Amy Saupe, Ginette Dobbins, Hillary A. Booth, Christianne Biggs, Katie Garman, Amy M. Woron, Umesh D. Parashar, Jan Vinjé, Aron J. Hall

**Affiliations:** Centers for Disease Control and Prevention, Atlanta, Georgia, USA (E. Leshem, M. Wikswo, L. Barclay, U.D. Parashar, J. Vinjé, A.J. Hall);; Ohio Department of Health, Columbus, Ohio, USA (E. Brandt, W. Storm, E. Salehi);; Wisconsin Department of Health Services, Madison, Wisconsin, USA (T. DeSalvo);; Wisconsin State Laboratory of Hygiene, Madison (T. Davis);; Minnesota Department of Health, St. Paul, Minnesota, USA (A. Saupe, G. Dobbins);; Oregon Public Health Division, Portland, Oregon, USA (H.A. Booth, C. Biggs);; Tennessee Department of Health, Nashville, Tennessee, USA (K. Garman, A.M. Woron)

**Keywords:** norovirus, GII.4 Sydney strain, outbreak, surveillance, viruses, enteric infections, United States

## Abstract

During 2012, global detection of a new norovirus (NoV) strain, GII.4 Sydney, raised concerns about its potential effect in the United States. We analyzed data from NoV outbreaks in 5 states and emergency department visits for gastrointestinal illness in 1 state during the 2012–13 season and compared the data with those of previous seasons. During August 2012–April 2013, a total of 637 NoV outbreaks were reported compared with 536 and 432 in 2011–2012 and 2010–2011 during the same period. The proportion of outbreaks attributed to GII.4 Sydney increased from 8% in September 2012 to 82% in March 2013. The increase in emergency department visits for gastrointestinal illness during the 2012–13 season was similar to that of previous seasons. GII.4 Sydney has become the predominant US NoV outbreak strain during the 2012–13 season, but its emergence did not cause outbreak activity to substantially increase from that of previous seasons.

Noroviruses (NoVs) are the most common cause of epidemic gastroenteritis worldwide and the leading cause of foodborne outbreaks in the United States (*1*–*3*). In the United States, NoVs cause 19–21 million illnesses and lead to 56,000–70,000 hospitalizations and 570–800 deaths each year (*4*). Severe disease associated with NoV occurs most frequently among older adults, young children, and immunocompromised patients (*4*–*7*). NoV outbreaks occur year round, but activity increases in the United States during the winter months; 80% of reported outbreaks occur during November–April (*8*,*9*).

NoVs belong to the family *Caliciviridae* and can be grouped into at least 5 genogroups (GI–GV), which are further divided into at least 35 genotypes (*2*,*10*). Most NoV outbreaks are attributed to genotype GII.4, which evolve rapidly over time (*11*,*12*). During the past decade, new GII.4 strains have emerged every 2–3 years and replaced previously predominant GII.4 strains. The emergence of new NoV strains is believed to be related, in part, to the antigenic diversity of the novel strain that leads to at least partial escape from preexisting herd immunity acquired against the predominant circulating strain (*12*). These new NoV strains have often, but not always, led to increased outbreak activity (*8*,*10*,*13*,*14*).

In March 2012, a new GII.4 NoV strain was identified in Australia. Named GII.4 Sydney, this emergent strain has since caused acute gastroenteritis outbreaks in New Zealand, Japan, Western Europe, and Canada (*15*–*17*). Preliminary indicators of increased NoV outbreak activity, including an increase in the number of confirmed cases and hospital-related outbreaks in late 2012, were presumed to be associated with emergence of GII.4 Sydney in several of those countries (*15*,*17*,*18*). In the United States, GII.4 Sydney became the predominant NoV strain implicated in outbreaks during the last 4 months of 2012 (*19*).

To assess whether the emergence of GII.4 Sydney strain was associated with an increase in overall NoV disease activity in the United States, we examined data from NoV outbreaks in 5 states and emergency department visits for gastrointestinal illness in 1 state during the 2012–13 season and compared these data with those of the 2 previous seasons. Furthermore, we compared epidemiologic (e.g., setting and mode of transmission) and clinical features of patients in outbreaks attributed to GII.4 Sydney with those of outbreaks attributed to other strains during the 2012–13 season.

## Methods

### NoV Outbreak Data and Analysis

The Centers for Disease Control and Prevention (CDC) operates 2 surveillance systems for NoV outbreaks in the United States—the National Outbreak Reporting System (NORS) and CaliciNet. NORS is a comprehensive reporting system for all enteric disease outbreaks in the United States, regardless of cause or transmission mode (*9*). NORS data include general outbreak characteristics, patient demographics, symptoms, and clinical outcomes. CaliciNet is an electronic laboratory surveillance network that collects information on genetic sequences of NoVs implicated in outbreaks (*11*). CaliciNet laboratories perform molecular typing of NoV strains by using standardized laboratory protocols for reverse transcription PCR followed by sequence analysis. Sequence data are then uploaded into a central database to monitor national trends in circulating NoV strains. Information about outbreaks is often reported by state health departments to CDC several months after outbreaks occur; therefore, both NORS and CaliciNet data may be subject to substantial reporting delays.

Beginning in August 2012, a network of 5 sentinel states was established to improve the timeliness of NoV outbreak reporting through NORS and CaliciNet and thereby allow near real-time assessment of NoV activity. These 5 states (Minnesota, Ohio, Oregon, Tennessee, and Wisconsin) include ≈33 million residents or 11% of the total US population spread across several regions of the country (*20*). In addition, these 5 states had historically the highest per capita reporting rates for NoV outbreaks and therefore were least likely to be affected by underreporting biases. State health departments that participate in this network–the Norovirus Sentinel Testing and Tracking (NoroSTAT) network–report suspected NoV outbreaks through NORS and CaliciNet within 7 business days of notification of the outbreak to the state health department. NoroSTAT reporting allows NoV strain data uploaded through CaliciNet to be rapidly linked with epidemiologic characteristics of outbreaks reported through NORS by using consistent outbreak identifiers in each system. Aside from more timely and complete data reporting, outbreak reporting practices and case definitions among NoroSTAT participants otherwise remained unchanged.

The present study was restricted to data reported to NORS and CaliciNet by NoroSTAT states on all confirmed and suspected NoV outbreaks with first illness onset dates during August 1, 2012–April 16, 2013. To compare the level of outbreak activity in the 2012–13 season with that in previous years, we extracted data for the same time during the 2 previous seasons (August 1, 2011–April 16, 2012 and August 1, 2010–April 16, 2011) from NORS for the NoroSTAT states. A confirmed NoV outbreak is defined as >2 cases of similar enteric illness associated with a common exposure that are laboratory confirmed for NoV by reverse transcription PCR, enzyme immunoassay, or electron microscopy. A suspected NoV outbreak is defined as >2 cases of similar enteric illness associated with a common exposure in which NoV was the suspected causative agent, which is determined by each reporting site (e.g., supportive clinical or epidemiologic information or outbreaks with only 1 positive NoV specimen).

NoV genotype and sequence data were extracted from CaliciNet, and epidemiologic data, including demographics, symptoms, and outcome data, were extracted from NORS. Season onset was defined as the week by which at least 10% of the total number of NoV outbreaks during August 1–April 16 of each year had occurred. Seasonal peak in NoV outbreak activity was defined as the month with the highest number of NoV outbreaks. Season duration was defined as the number of weeks between which 10% and 90% of the total number of NoV outbreaks had occurred.

For comparison of GII.4 Sydney outbreak characteristics with those attributed to other NoV strains, outbreaks for which the NoV strain was reported through CaliciNet were grouped into 2 categories: GII.4 Sydney and non–GII.4 Sydney. A third category consisted of outbreaks reported by NoroSTAT states only through NORS (i.e., for which strain data were not available). Data on demographic characteristics, symptoms, and clinical outcomes were not available from all outbreaks. To minimize potential biases from underreporting, we included demographic characteristics, symptoms, and outcomes and analyzed them only when available for >10% of reported illnesses across all reported outbreaks.

To test the increase in proportion of NoV outbreaks attributed to the GII.4 Sydney strain by month during the 2012–13 season, we used the χ^2^ test for trend. Categorical variables were compared by calculating rate ratios with 95% CIs. Statistical significance was set at p<0.05 by using the mid-*p* exact test. Analyses were performed by using SAS v9.3 (SAS Institute Inc., Cary, NC, USA).

### Syndromic Surveillance Data and Analysis

Ohio collects statewide syndromic surveillance data from 178 emergency department and urgent care facilities (referred to collectively herein as emergency departments) into a system called EpiCenter. This system is used by state and local public health agencies to detect, track, and characterize health events, such as pandemic influenza, outbreaks, environmental exposures, and potential bioterrorism, in real-time. The system gathers information on patient symptoms with identification removed and automatically alerts public health officials when an unusual pattern or trend is occurring. We used EpiCenter data for August 1, 2010–April 16, 2013, to measure the weekly proportion of patients with gastrointestinal syndrome, defined as the chief complaint that included the following symptom keywords: abdominal, diarrhea not watery/bloody, diarrhea watery/bloody, nausea, and vomiting. To assess the correlation between the proportion of emergency department visits attributed to gastrointestinal illness and the number of NoV outbreaks, we used the Pearson correlation coefficient.

## Results

### Norovirus Outbreak Data

**Figure 1:**
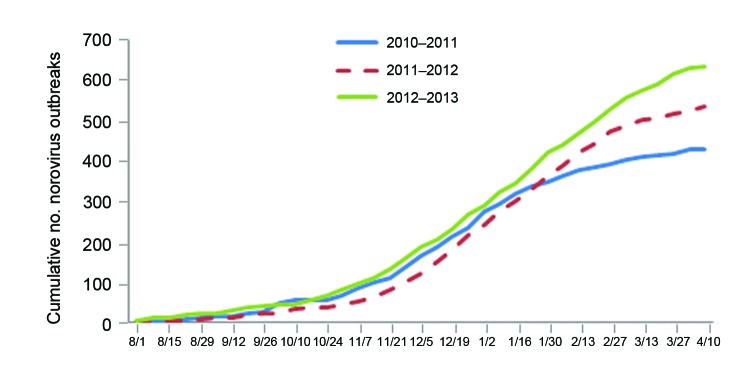
Number of suspected and confirmed norovirus gastroenteritis outbreaks by week of illness onset: Minnesota, Ohio, Oregon, Tennessee, and Wisconsin, August 1, 2010–April 16, 2013 (no. outbreaks = 1,605).

During August 1, 2012–April 16, 2013, a total of 637 NoV outbreaks were reported by Minnesota, Ohio, Oregon, Tennessee, and Wisconsin. Season onset was similar in 2012–13 compared with 2011–12 and 2010–11 (October 24 vs. November 7 and October 3, respectively, Figure 1). By April 16, the cumulative number of outbreaks was slightly higher in the 2012–13 season than in the 2011–12 and 2010–11 seasons (637 vs. 536 and 432, respectively). However, the data varied considerably between the states. The cumulative number of outbreaks increased in 3 states (Oregon, Tennessee, and Ohio), Wisconsin data were similar for each season, and Minnesota reported a decreased cumulative number of outbreaks (Technical Appendix Figure). Peak outbreak activity occurred in January 2013 (152 outbreaks) and was 16% higher than the average of peak month outbreak activity in the 2 preceding seasons (148 outbreak in January 2012 and 113 outbreaks in January 2011). Season duration was 21 weeks in 2012–13, compared with 18 weeks in 2011–12 and 22 weeks in 2010–11.

**Figure 2:**
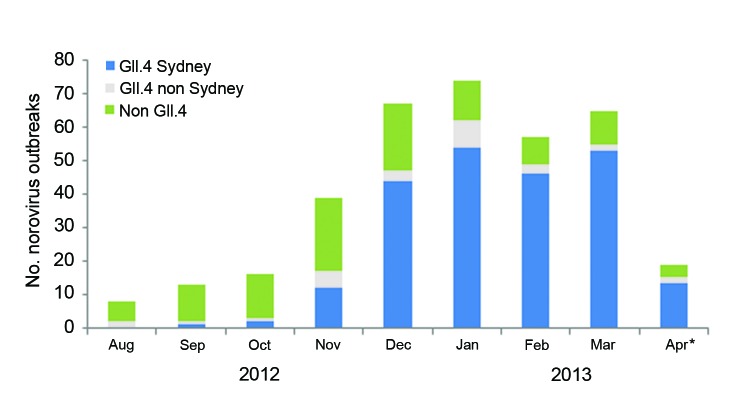
Genotypes of confirmed norovirus gastroenteritis outbreaks in Minnesota, Ohio, Oregon, Tennessee, and Wisconsin, August 1, 2012–April 16, 2013 (no. outbreaks = 358). *Data available for outbreaks during April 1, 2013–April 16, 2013.

Sequence data were available for 358 (56%) of the 637 outbreaks. Outbreaks with sequence data were more likely to have reported the mode of transmission as foodborne, and transmission was more likely to have occurred at restaurants and less likely to have occurred at schools (data not shown). Other characteristics were similar between the outbreaks with and without sequence data. Of these, 226 (63%) were attributed to the GII.4 Sydney strain. The proportion of outbreaks attributed to GII.4 Sydney increased from 8% in September 2012 to 82% in March 2013 (Figure 2, χ^2^ test for trend; p<0.001). In December 2012, GII.4 Sydney became the predominant strain, accounting for 44 (66%) of the 67 outbreaks reported that month from which sequence data were available. GII.4 Sydney accounted for most sequenced outbreaks in all 5 states: 16 (57%) of 28 in Minnesota, 50 (56%) of 89 in Ohio, 57 (61%) of 94 in Oregon, 27 (73%) of 37 in Tennessee, and 76 (69%) of 110 in Wisconsin. The proportion of GII.4 Sydney outbreaks in the states with an increased cumulative number of outbreaks in 2012–2013 was not significantly different from the proportion in those states without an increased number of outbreaks (61% vs. 67%, respectively; p = 0.27).

Overall, the most commonly identified mode of transmission was person-to-person, which occurred in 481 (75.5%) of 637 outbreaks (Table 1). The proportions of different modes of transmission were similar among GII.4 Sydney and non–GII.4 Sydney outbreaks. Healthcare settings were reported most frequently across all outbreaks. However, GII.4 Sydney outbreaks occurred more frequently in healthcare settings (long-term care facilities and hospitals) than did non–GII.4 Sydney outbreaks (75.2% vs. 47.7%, respectively; rate ratio [RR] 1.58; 95% CI 1.30–1.91). GII.4 Sydney outbreaks occurred less frequently in schools and childcare centers than did non–GII.4 Sydney outbreaks (1.8% vs. 21.2%, respectively; RR 0.08; 95% CI 0.03–0.23). The proportion of outbreaks that occurred at restaurants or banquet facilities was similar for GII.4 Sydney and non–GII.4 Sydney outbreaks. Outbreaks for which sequence data were available were similar with respect to setting and transmission mode to those without sequence data.

**Table 1:** Number and percentage of norovirus gastroenteritis outbreaks, by genotype, strain, setting, and mode of transmission in Minnesota, Ohio, Oregon, Tennessee, and Wisconsin, August 2012–April 2013*

Outbreak	No. (%) outbreaks with sequence data			No. (%) outbreaks with no sequence data, n = 279	Total no. (%), N = 637
	GII.4 Sydney, n = 226	Non–GII.4 Sydney, n = 132	RR (95% CI)		
Mode of transmission					
Person to person	172 (76.1)	93 (70.5)	1.08 (0.95–1.23)	216 (77.4)	481 (75.5)
Foodborne	35 (15.5)	28 (21.2)	0.73 (0.47–1.14)	28 (10.0)	91 (14.3)
Water	0	1 (0.8)	NA	0	1 (0.2)
Environmental	0	2 (1.5)	NA	0	2 (0.3)
Other/unknown	19 (8.4)	8 (6.1)	1.39 (0.62–3.08)	35 (12.5)	62 (9.7)
Setting					
LTCF/hospital	170 (75.2)	63 (47.7)	1.58 (1.30–1.91)	189 (67.7)	422 (66.2)
School/CCC	4 (1.8)	28 (21.2)	0.08 (0.03–0.23)	49 (17.6)	81 (12.7)
Restaurant/banquet facility	35 (15.5)	25 (18.9)	0.82 (0.51–1.30)	23 (8.2)	83 (13.0)
Other/multiple settings†	14 (6.2)	13 (9.8)	0.63 (0.31–1.30)	15 (5.4)	42 (6.6)
Unknown	3 (1.3)	3 (2.3)	0.58 (0.12–2.85)	3 (1.1)	9 (1.4)

*RR, rate ratio; NA, not available; LTCF, long- term care facility; CCC, childcare center. †Other settings include the following: private home (n = 15), noncafeteria workplace (n = 7), church (n= 2), other religious location (n = 2), hotel (n = 2), party (n = 3), apple orchard (n = 1), camp (n = 1), football tournament (n = 1), ship (n = 1), indoor playground (n = 1), movie theater (n = 1), trip to relatives (n = 1), other unspecified (n = 1), and multiple settings (n = 3).

Aggregate patient data were available for 310 (49%) of the 637 NoV outbreaks (9,018 patients); 104 of the outbreaks were GII.4 Sydney outbreaks, 74 were non–GII.4 Sydney outbreaks, and 132 were reported only through NORS without genotype data. The proportion of patients >50 years of age was higher in GII.4 Sydney outbreaks than in non–GII.4 Sydney outbreaks (65.9% vs. 43.9%, respectively; RR 1.50; 95% CI 1.39–1.62; Table 2).

**Table 2:** Number and percentage of patients in outbreaks of acute gastroenteritis attributed to norovirus, by symptoms, clinical outcomes, and reported strain in Minnesota, Ohio, Oregon, Tennessee, and Wisconsin, August 2012–April 2013*

Characteristic	No. (%) patients linked to outbreaks with sequence data			No. (%) patients linked to outbreaks with no sequence data	No. (%) total
	GII.4 Sydney	Non–GII.4 Sydney	RR (95% CI)		
Demographics					
Sex					
M	576 (31.8)	448 (33.4)	0.95 (0.86–1.06)	355 (31.8)	1,379 (32.3)
F	1,235 (68.2)	895 (66.6)	1.02 (0.97–1.08)	761 (68.2)	2,891 (67.7)
Age, y					
0–4	14 (0.9)	26 (2.3)	0.42 (0.22–0.79)	39 (3.9)	79 (2.2)
5–9	5 (0.3)	156 (13.7)	0.02 (0.01–0.06)	120 (12.0)	281 (7.8)
10–19	109 (7.4)	86 (7.5)	0.98 (0.74–1.28)	107 (10.7)	302 (8.3)
20–49	376 (25.4)	372 (32.6)	0.78 (0.69–0.88)	190 (19.0)	938 (25.9)
50–74	310 (20.9)	174 (15.2)	1.37 (1.16–1.63)	157 (15.7)	641 (17.7)
>75	666 (45.0)	327 (28.7)	1.57 (1.41–1.75)	387 (38.7)	1,380 (38.1)
Symptoms					
Diarrhea	2,385 (84.8)	1,549 (75.7)	1.12 (1.09–1.15)	1,882 (81.6)	5,816 (81.2)
Vomiting	1,337 (53.0)	1,214 (60.4)	0.88 (0.83–0.92)	1,356 (60.9)	3,907 (57.8)
Fever	1,191 (50.9)	1,126 (58.7)	0.87 (0.82–0.92)	1,030 (56.8)	3,347 (55.1)
Abdominal cramps	995 (48.1)	850 (54.5)	0.88 (0.83–0.94)	544 (44.9)	2,389 (49.3)
Outcome					
Outpatient visit	122 (7.9)	55 (4.3)	1.81 (1.33–2.47)	54 (5.9)	231 (6.2)
Emergency department visit	43 (2.4)	24 (1.8)	1.23 (0.79–2.13)	34 (3.7)	101 (2.5)
Hospitalized	49 (2.2)	22 (1.4)	1.54 (0.93–2.53)	65 (4.4)	136 (2.6)
Death	9 (0.4)	3 (0.2)	2.2 (0.60–8.12)	9 (0.4)	21 (0.3)

*Because of the preliminary nature of the data, information on demographic characteristics, symptoms, or outcomes were available for 310 (49%) of 637 total norovirus outbreaks reported during the study period. The number of patients and the relative proportions of illnesses by symptoms and outcomes from the outbreaks that included such data are reported. RR, rate ratio.

Patients from GII.4 Sydney outbreaks reported diarrhea slightly more frequently than patients from outbreaks associated with non–GII.4 Sydney viruses (84.8% vs 75.7%, respectively; RR 1.12; 95% CI 1.09–1.15; Table 2). In contrast, vomiting, fever, and abdominal cramps were reported by a lower proportion of patients from GII.4 Sydney outbreaks than those from non–GII.4 Sydney outbreaks (Table 2).

A higher proportion of patients required outpatient visits among those affected by GII.4 Sydney outbreaks than among those in non–GII.4 Sydney outbreaks (7.9% vs. 4.3%, respectively; RR 1.81; 95% CI 1.33–2.47). We observed 9 (0.4%) deaths among 2,324 patients associated with GII.4 Sydney outbreaks for whom we had mortality data, compared with 3 (0.2%) deaths reported among 1,706 patients associated with non–Sydney outbreaks (RR 2.20, 95% CI 0.60–8.12; Table 2). Hospitalization and emergency department visits occurred at similar proportions in GII.4 Sydney and non–GII.4 Sydney outbreaks.

### Syndromic Surveillance Data

**Figure 3:**
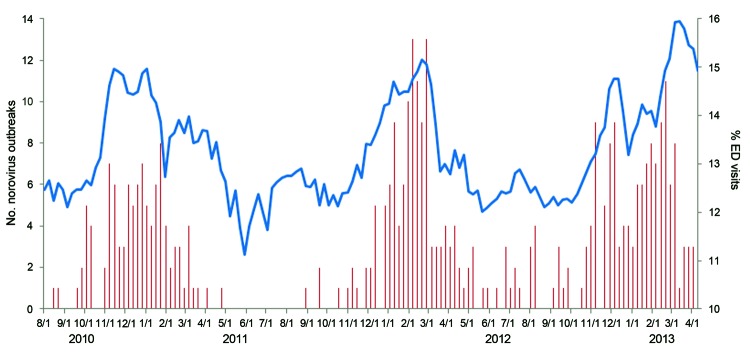
Percentage of emergency department and urgent care (ED) visits for gastrointestinal illness as reported through the Ohio EpiCenter syndromic surveillance system and number of suspected and confirmed norovirus gastroenteritis outbreaks by week, August 1, 2010–April 16, 2013.

On average, the proportion of total weekly emergency department visits reported through Ohio EpiCenter that were classified into the gastrointestinal syndrome category was 13.3% (median 12.9%, range 11.1%–15.9%; Figure 3). The proportion of weekly gastrointestinal emergency department visits was strongly correlated with NoV outbreaks reported from Ohio (Pearson correlation coefficient; r = 0.68). The magnitude and timing of seasonal increase in the proportion of emergency department visits for gastrointestinal syndrome during the 2012–13 season were similar to those of previous seasons.

## Discussion

The global emergence of a novel GII.4 NoV strain (GII.4 Sydney) in late 2012 prompted concern about a possible increase in incidence and severity of NoV disease and outbreaks. In the United States, GII.4 Sydney has become the predominant strain as the 2012–13 season has progressed, replacing the previously predominant GII.4 New Orleans strain (*11*). However, the emergence of GII.4 Sydney does not appear to have caused a substantial increase in the level of outbreak or endemic NoV disease activity compared with the previous 2 seasons.

Scotland and Denmark, like the United States, have reported the emergence of GII.4 Sydney as the predominant cause of NoV outbreaks during October–November 2012 (*16*,*17*). In Scotland and the United Kingdom, the onset of the 2012 winter NoV season was reported to be earlier than usual (*17*,*21*). Earlier descriptions of the GII.4 Sydney strain’s emergence in other countries suggested a possible increase in the level of NoV activity compared with that of previous seasons (*15*,*18*). In our study, we found increased peak monthly levels of outbreak activity and cumulative numbers of outbreaks during the 2012–13 season compared with the 2 previous seasons. We also found higher cumulative numbers of outbreaks in 3 of the 5 reporting states during the 20012–13 season. However, these increases were not related to higher proportions of GII.4 Sydney outbreaks when findings were compared with results from states that had no increase in outbreak activity this season. Thus, the observed increase in outbreak activity during the 2012–13 season likely represents random seasonal and state variation rather than a direct result of the emergence of GII.4 Sydney. This conclusion is further supported by syndromic surveillance data from Ohio, which did not show an increase in the proportion of gastrointestinal illness among emergency department patients compared with proportions in previous seasons.

Most outbreaks attributed to the GII.4 Sydney strain occurred in healthcare–related settings and were predominantly transmitted from person to person; these findings were similar to previous data for outbreaks attributed to other GII.4 NoV strains (*10*,*22*). This finding could be caused by age-associated biologic difference in virus infectivity, other virus and host-related factors, or relatively better outbreak reporting in long-term care facilities than in other settings. Additional studies are needed to better define the basis for this observed difference.

Although aggregate patient data were available for less than half of the outbreaks and primarily from those that occurred earlier in the season, some preliminary trends in the affected populations and clinical profiles were observed. GII.4 Sydney outbreaks disproportionally affected older persons, consistent with the observed predilection toward long-term care facilities. Patients affected by GII.4 Sydney were more likely to have diarrheal illness and less likely to have vomiting, fever, and abdominal cramps. Age-associated patterns of illness may explain this observation; however, it differs from a previous study that concluded that vomiting, fever, and abdominal cramps are more prevalent in patients infected with GII.4 NoV strains than those infected with non–GII.4 strains (*23*). We found higher rates of outpatient visits among patients infected with the GII.4 Sydney strain than among those infected with strains other than GII.4 Sydney, most of whom were infected with non-GII.4 strains. This finding is consistent with the previously reported association between GII.4 strains and severe outcomes (*24*). However, since hospitalization and emergency department visits occurred at similar rates, and given the difference in age of patients in GII.4 Sydney and non–GII.4 Sydney outbreaks, we were unable to associate GII.4 Sydney with differences in clinical severity.

Study limitations include the lack of data from a complete year of GII.4 Sydney transmission and inclusion of data from only 5 states, which may not be representative of national NoV outbreak activity. To confirm our findings, subsequent analysis of complete NORS and CaliciNet data from all states is necessary when it becomes available. Nevertheless, by using the currently available data reported through NoroSTAT, we ensured stable NoV outbreak definitions and reporting, and timely data allowed us to rapidly assess the magnitude of the current season. Moreover, NoroSTAT currently covers >10% of the US population from states with high per-capita outbreak reporting spread across several geographic regions; thus, it likely serves as a reasonable sentinel system for assessment of overall US NoV activity and the effects of emergent strains. Reporting results of primarily early-season aggregate patient data (demographic, symptom, and clinical outcomes) may introduce bias because these data are likely confounded by outbreak setting, which shifts toward a higher proportion of healthcare-associated outbreaks in the winter. Outbreak identification and reporting may differ across settings, and therefore, genotypes occurring in settings with lower reporting rates may be underrepresented in the analyses. In addition, including suspected NoV outbreaks may have resulted in slightly lower specificity of case definitions; however, the probability of NoV as causative agent is high when no other causative agent is identified because NoV is the most common cause of enteric disease outbreaks (*1*). Last, the definition of suspected NoV infection may have differed between NoroSTAT sites; however, all sites have confirmed stable and standardized outbreak definitions during the study period (2010–2013), which validates comparisons with previous seasons.

In conclusion, GII.4 Sydney has rapidly emerged to become the predominant outbreak strain in the United States; however , timely outbreak surveillance data collected through NORS and CaliciNet and syndromic emergency department data did not indicate that GII.4 Sydney caused a substantial increase in NoV activity during the 2010–13 season compared with the previous 2 seasons. Further analysis of NoV outbreaks reported through NORS and CaliciNet, as well as data on endemic NoV disease, can help verify the strain-specific differences in clinical profile of NoV disease observed in this preliminary assessment. Continuing to expand enhanced real-time outbreak reporting and syndromic surveillance will enable the magnitude and severity of emergent NoV strains, such as GII.4 Sydney, to be evaluated quickly.

Technical Appendix Number of suspected and confirmed norovirus gastroenteritis outbreaks by week of illness onset and by reporting state.
