# Supplementary material for: Effects and Clinical Significance of GII.4 Sydney Norovirus, United States, 2012–2013
Source: Emerg Infect Dis. 2013 Aug;19(8):1231–8. doi: 10.3201/eid1908.130458 (PMC3739516; doi:10.3201/eid1908.130458)
Supplement: Technical Appendix — Number of suspected and confirmed norovirus gastroenteritis outbreaks by week of illness onset and by reporting state. [file 13-0458-Techapp-s1.pdf]

Article DOI: http://dx.doi.org/10.3201/eid1908.130458

Effects and Clinical Significance of GII.4 Sydney Norovirus, United States, 2012–2013

Technical Appendix

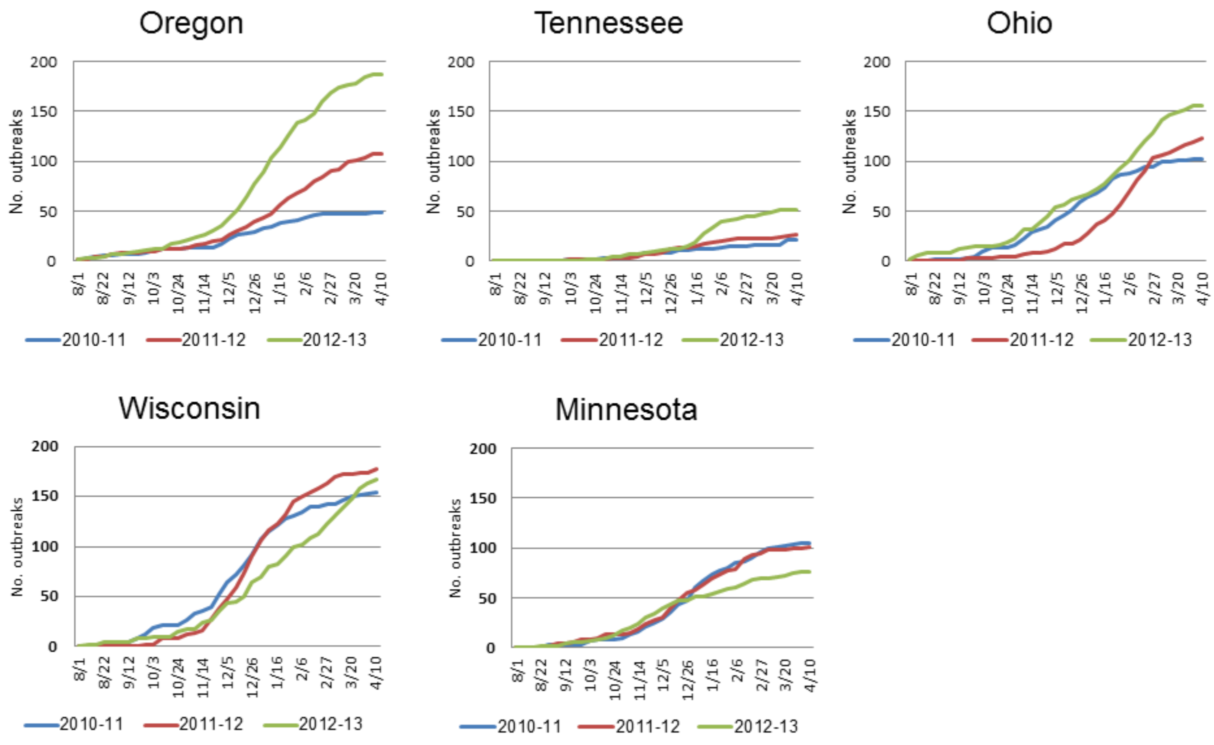

Technical Appendix Figure. Number of suspected and confirmed norovirus gastroenteritis outbreaks by week of illness onset and by reporting state: Oregon (no. outbreaks = 344), Tennessee (no. outbreaks = 99), Ohio (no. outbreaks = 381), and Wisconsin (no. outbreaks = 499), Minnesota (no. outbreaks = 282), 2010–2013.
